# Academic Career Progression of Chinese-Origin Pharmacy Faculty Members in Western Countries

**DOI:** 10.3390/pharmacy6040104

**Published:** 2018-09-21

**Authors:** Weixiang Zhang, Hao Zhong, Yitao Wang, Ging Chan, Yuanjia Hu, Hao Hu, Defang Ouyang

**Affiliations:** State Key Laboratory of Quality Research in Chinese Medicine, Institute of Chinese Medical Sciences (ICMS), University of Macau, Taipa, Macau, China; 13980967387@163.com (W.Z.); MB75822@umac.mo (H.Z.); YTWang@umac.mo (Y.W.); gchan@umac.mo (G.C.); YuanjiaHu@umac.mo (Y.H.); HaoHu@umac.mo (H.H.)

**Keywords:** underrepresented minorities, Chinese-origin faculty, academic career progression, academic, pharmacy

## Abstract

**Background:** The field of Pharmacy education is experiencing a paucity of underrepresented minorities (URMs) faculty worldwide. The aim of this study is to investigate the current professional status of Chinese-origin pharmacy faculty members, who are considered as a good model of URMs at pharmacy academia in western countries, and identify the influencing factors to their academic career progression in academic careers. **Methods:** An online questionnaire was sent to Chinese-origin academic staffs at pharmacy schools in US, UK, Canada, Australia, and New Zealand. The survey comprised demographic information, educational background, and the influencing factors to academic career progression. **Results:** The vast majority of Chinese faculty members who worked in US were male. Individuals with junior academic title comprised the largest proportion. Over 75% of Chinese-origin pharmacy academics were involved in scientific disciplines (e.g., pharmaceutics, pharmacology, and medicinal chemistry). Usually, Chinese-origin academic members spent 4 years obtaining their first academic jobs after finishing PhD degree, and need 5–6 years to get academic promotion. The contributing factors of academic promotion were high quality publications and external funding. **Conclusion:** Our research offers a deep insight into academic career progression for URMs and give some valuable advice for their pharmacy academic paths.

## 1. Introduction

With the development of globalization impacting education, western countries with English-speaking culture have attracted many scholars from non-English speaking countries [1]. One report by the National Center for Education Statistic (NCES) indicated that there was a total of 761,619 full-time faculty members at U.S degree-granting institutions in 2011. Among them, 79.3% of faculty members were White, while underrepresented minorities (NRMs) had reached to 20.7% (Black, Hispanic, Asian, Pacific Islander, and American Indian/Alaska Native). Asians showed the most obvious increase, which was from only 7.6% in 2007 to 8.8% in 2011 [2]. Academic institutions are challenged to increase the proportion of these NRMs faculty, which are more likely to suffer from injustice in faculty promotion [3], such as limited network opportunities, confronting bias and stereotypes, and lack of ethnic role models and mentors [4,5]. China has grown up the largest economy and population in the Asian and Chinese scholars working in the western counties are performing an obvious upward trend and becoming a non-neglectful group, so attention urgently needs to be taken for Chinese faculty members.

Pharmacy education in the US was experiencing challenges about the ratio of URM students [6]. A recent study showed that the composition of URM pharmacy faculty members (10% approximately) was much less than minority representation in the general American population, and there was very little growth in the number of URM pharmacy students and faculty members from 1989 to 2009 [7]. Nevertheless, the situation in UK pharmacy looked different from US pharmacy. A 2012 study investigated the ethnic composition of first-year pharmacy students at Aston pharmacy in the UK. The Aston pharmacy program has a history of over 100 years and among the ranks top 5 British pharmacy schools according to University Subject Table 2011 for pharmacy and pharmacology [8]. In this research, it was found that over 70% of first-year students at Aston pharmacy school originated from Asian counties, while white students were less than 10% [9]. American academic institutions should make effort to overcome these obstacles to make faculty workforce diverse.

At present, some researches about the academic career progression of URMs have been published. The aim of SE Kaplan et al.’s study [10] was to understand differences in productivity, advancement, retention, satisfaction, and compensation comparing underrepresented medical (URM) faculty with other faculty at multiple institutions. The result showed that no differences were identified in federal grant acquisition, senior leadership roles, career satisfaction, or compensation between URM and white faculty. Price EG et al. found racial differences led to disparities in qualifications for training programs and subsequent career path to faculty positions [5]. It revealed that some structural factors could hinder the recruitment and career advancements for URM faculty members. Other studies reported that major ethnic groups in pharmacy applicant pools were associated with higher grade point averages (GPAs) and pharmacy college admission test (PCATs) scores, and more likely to get into pharmacy colleges [11].

Currently, there is no quantitative study to investigate academic career progression of racial minority pharmacy faculty members. Chinese faculty, which constitute an important component of URM, can be considered as a good model of the minorities at pharmacy academia. Thus, the aim of this study was to investigate professional current status and the influencing factors of Chinese-origin pharmacy faculty members in western countries, and provide some advice for URMs.

## 2. Methodology

The British academic system is ranked from lecturer, senior lecturer, reader to professor, while the American system included assistant professor, associate professor, and professor. In this study, academic staff were identified, including lecturers/assistant professors, senior lecturers/associate professors, and readers/professors in the university. “Chinese-origin” referred to the ethnic Chinese who have received undergraduate education in China. “Chinese-origin” faculty members were investigated as URMs.

### 2.1. Research Instrument

An anonymous on-line questionnaire was used in the research that we carried out to investigate current academic career progression status of Chinese-origin faculty members. The questionnaire consisted of 20 questions and could be divided into three parts.

#### 2.1.1. Demographic Information

In the first section of the questionnaire, a short introductory text about the intent of the questionnaire was presented followed by demographic information about Chinese-origin faculty: name, sex, age, country, institution, and academic discipline. Academic discipline contained pharmaceutics, pharmacology, medicinal chemistry, Clinic pharmacy (or pharmacy practice), pharmacy administration, and others.

#### 2.1.2. Education Background

In the second section of the questionnaire, the contents mainly include two parts: the participants’ major and professional degrees (bachelor, master, and PhD degree, respectively). 

#### 2.1.3. Career Development

In the third section of questionnaires, the aim was to obtain the information about the development of academic careers for participants, including academic ranks and influencing factors to the academic promotion. These influencing factors included high-quality publications, education background, research area, language skills, honors and awards, teaching, service, PhD supervision, recognition from peers, external fund supporting, and network. These questions were based on a 5-point Likert scale (“Very important”, “Moderately important”, “Slightly important”, “Low important”, “Not important”). The last question was an open-ended question about suggestions for Chinese pharmacy education.

### 2.2. Participants

At present, western countries are mainly of comprised the European Union member states, the United States, Canada, Australia, New Zealand, and parts of Latin America. After investigation of the status of pharmacy faculty members in these counties, it was found that most of Chinese-origin pharmacy faculty members were in pharmacy schools of five western countries: US, UK, Canada, Australia, and New Zealand. Thus, 331 participants were selected from the accredited pharmacy in these five countries. Contact E-mails of participants were obtained from the website of schools, as well as the biography, name and photograph of academic staffs. The project had been approved by University of Macau (UM) Ethic Committee.

### 2.3. Data Analysis

An online survey (Google Form) was sent to each faculty member separately. The responses information was collected by Google Form. Data statistics were analyzed using IBM SPSS Statistics version 19.0 (IBM, Chicago, IL, USA). The prior significance level was set at 0.05.

## 3. Results

### 3.1. Professional Status of Current Chinese-Origin Pharmacy Academics

**Figure 1:**
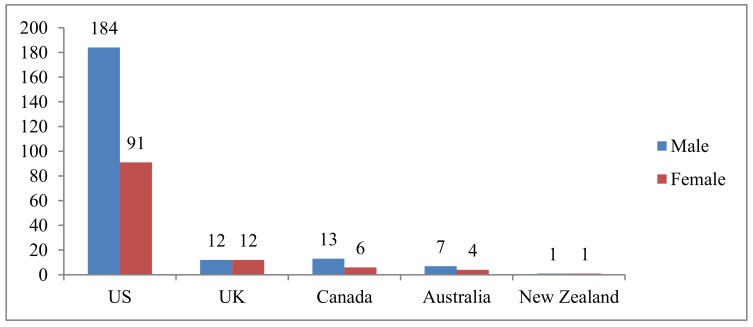
Distribution of Chinese-origin faculty members by the gender and country (N = 331).

Online surveys were sent to 331 Chinese-origin pharmacy faculty members and 59 effective responses were collected. The response rate was 18.8%. Figure 1 showed the country distribution and gender of Chinese-origin pharmacy faculty. The vast majority of Chinese faculty members (275) were in US, accounting for 83% of total workforce (N = 331). A total of 66% of Chinese-origin pharmacy faculty members were male, while female was only approximately one third.

Table 1 indicates the distribution of each academic rank among these five countries. It was clearly shown that junior faculty members (assistant professor in American system or lecturer in British system) comprised the largest proportion (40.5%), followed by middle-level academics with 32.3% (associate professor in American system or senior lecturer in British system), while only 27.2% were senior faculty members (such as reader and professor).

Table 2 clearly shows that the distribution of Chinese-origin faculty members by discipline and country. Most Chinese-origin faculty members were involved with the scientific disciplines, such as pharmaceutics (26.9%), pharmacology (30.5%), and medicinal chemistry (19.1%), while only 16.6% of them majored in clinical pharmacy and 6.9% in pharmacy administration.

### 3.2. Academic Career Progression in Pharmacy Academia of Chinese Faculty Members

Table 3 indicates the responded statistics (*n* = 59) for academic promotions of Chinese faculty members in their academic careers. Usually Chinese-origin academic members spent approximately 4 years to obtain their first academic jobs after finishing PhD degree. Moreover, it took them nearly 5–6 years to get academic promotion at each academic level.

**Figure 2:**
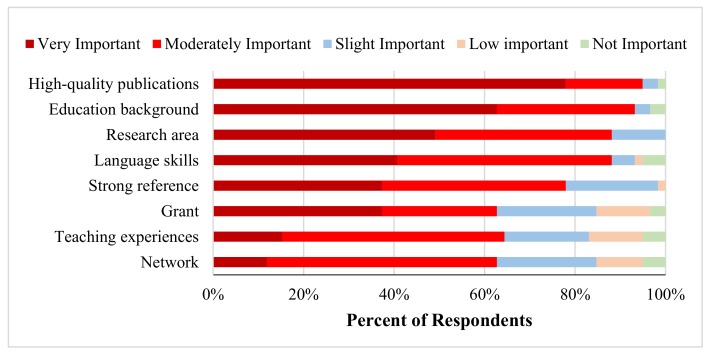
Ranking of contributing factors by importance in the first academic jobs for Chinese origin faculty (*n* = 59).

Figure 2 indicates the importance ranking of the contributing factors to obtain the first academic jobs. The top three important factors were high quality publications, educational background, and research area. Language skills, strong reference, and grant were moderately important to getting academic jobs. In addition, several representative arguments were raised by some faculty members. For example, academic pedigree or work experiences in pharmaceutical industry was also important to getting the first academic jobs. 

**Figure 3:**
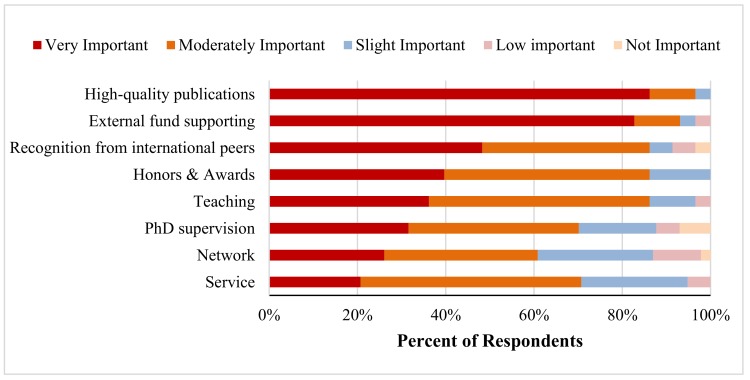
Ranking of contributing factors by importance to academic career progression for Chinese pharmacy faculty members (*n* = 59).

Figure 3 reveals the key factors to academic career progression for Chinese-origin pharmacy academics. It is not surprising that high quality publications and external fund support were two most important factors to their professional promotion. Recognition from international peers, honor/awards, teaching, and PhD supervision were also considered important to professional advancement. In addition, some respondents mentioned that generic skills, early occupation planning, and participation in school affairs and leadership were significant factors to academic career progression.

## 4. Discussion

The results showed that the number of Chinese-origin pharmacy faculty working in US comprised largest share, followed by UK, Canada, Australia, and New Zealand. One reason for this might be that the ranking is positively related to the number of accredited pharmacy schools (US: 134; UK: 26; Canada: 13; Australia: 18; New Zealand: 2). Another possible reason is that US is the first choice of higher education for Chinese students. One report showed that Chinese-origin students in 2016 had reached up to 550,000. About 60% of among these students applied to the US schools. The third explanation was that pharmacy education in US put more weight on faculty mentoring programs for future faculty development and academic achievement [12]. Over 70% of US pharmacy schools had faculty mentoring program as the significant component of faculty development and academic environment [13].

The majority of respondents majored in scientific disciplines (e.g., pharmaceutics, pharmacology and medicinal chemistry), while the number of members majoring in clinical pharmacy was less than 20%. This was quite different from the distribution of academic staff at pharmacy schools in western countries. Usually, the percentage of faculty members within clinical pharmacy accounted for over half of the whole pharmacy faculty workforce, which was significantly more than that of other science-based disciplines [14,15]. A possible reason for the big difference might be different mode of pharmacy education in China. In the past, the curriculum of Chinese pharmacy education focused mainly on chemistry courses, which was called “chemistry models”. However, past pharmacy students in China were short of biomedical and clinical skills and practice experiences [16]. In general, clinical-related training courses took over 2 years at most pharmacy schools in western countries, but clinical training in Chinese system was less than a half-year [17]. This was consistent with the viewpoint from a respondent: “Chinese pharmacy schools do not train professional pharmacists. This is strange. They should train both pharmacists and researchers” (one participant from Australia).

The study also investigated influencing factors for academic career progression. High-quality publication still played the most important role in the process of getting their first academic jobs and professional advancements, which reflected the academic level and research area directly Academic performance is the most critical factor for getting the first academic job, such as educational background, research area, and language skills. With the further promotion of the academic career, external fund support and recognition from international peers become important. Chinese-origin faculty members usually spend 4 years getting the first job, but need 5–6 years to get academic promotion. The results are roughly consistent with previous studies. The impact factors mainly comprised academic performance, personal learning, and social change [18]. Currently, only few studies mention that fund supporting is key factor to career advancement.

## 5. Conclusions

The current research investigated academic career progression of Chinese-origin pharmacy faculty members in western countries from the multiple angles. Most Chinese-origin pharmacy faculty members worked in science-related subject due to different pharmacy training models between western countries and China. It would take more time to get the academic promotions for URMs. Publication still is the key factor to academic career progression for URMs, but the factors of assessment are relatively varied for academic career progression, such as external founding support and PHD supervision. So, scholars should make an effort to improve the educational background and release the high-quality publications at early states. After getting the jobs in academia, scholars need to broaden their views, not only focus on high-quality publications, but they also struggle for external funding support. Our research offers a deep insight into academic career progression for URMs and some valuable advice to Chinese pharmacy education. In the study, online survey was adopted, but only 59 effective responses were collected. The sample is so small that there may be deviation in the results. In addition, there is a lack of a comparative group in this study.

In further studies, researchers can investigate the mental status of Chinese-origin faculty who confront with the pressure of academic career progression. At the same time, the further studies can also compare Chinese-origin with other racial faculty to identify whether there exists any racial bias in career progression.

## Figures and Tables

**Table 1:** Number of Chinese-origin faculty with different academic rank in each country (N = 331).

Academic Rank	No (%)
US	
Assistant Professor	114 (34.4)
Associate Professor	87 (26.3)
Professor	74 (22.4)
UK	
Lecturer	10 (3.0)
Senior Lecturer	12 (3.6)
Reader/Professor	2 (0.6)
Canada	
Assistant Professor	6 (1.8)
Associate Professor	5 (1.6)
Professor	8 (2.4)
Australia	
Lecturer	3 (0.9)
Senior Lecturer	2 (0.6)
Reader/Professor	6 (1.8)
New Zealand	
Lecturer	1 (0.3)
Senior Lecturer	1 (0.3)
Reader/Professor	0 (0)
Grand Total	
Assistant Professor/Lecturer	134 (40.5)
Associate Professor/Senior Lecturer	107 (32.3)
Professor/Reader	90 (27.2)

**Table 2:** Status of Chinese-origin faculty by discipline and country (N = 331).

Discipline	Country (%)					Grand Total
	US	UK	Canada	Australia	New Zealand	
Clinical Pharmacy/pharmacy practice	49 (14.8)	1 (0.3)	1 (0.3)	4 (1.2)	0 (0)	55 (16.6)
Medicinal Chemistry	52 (15.6)	9 (2.7)	0 (0)	2 (0.6)	1 (0.3)	63 (19.1)
Pharmaceutics	68 (20.5)	11 (3.3)	6 (1.8)	3 (0.9)	1 (0.3)	89 (26.9)
Pharmacology	89 (26.9)	2 (0.6)	9 (2.7)	1 (0.3)	0 (0)	101 (30.5)
Pharmacy Administration	18 (5.4)	1 (0.3)	3 (0.9)	1 (0.3)	0 (0)	23 (6.9)
Grand total	275 (83.1)	24 (7.3)	19 (5.7)	11 (3.3)	2 (0.6)	331 (100)

**Table 3:** Academic promotion of Chinese faculty members (*n* = 59).

Promotion	Mean Years Spending	SD *
From PhD to first academic job	4.3	2.2
From assistant professor/lecturer to associate professor/senior lecturer	5.7	1.5
From associate professor/senior lecturer to professor	5.1	1.8

SD * = Standard Deviation.

## References

[1] Jöns H., Hoyler M. (2013). Global geographies of higher education: The perspective of world university rankings. Geoforum.

[2] Snyder T.D., Dillow S.A. (2013). Digest of Education Statistics 2012.

[3] Fang D., Moy E., Colburn L., Hurley J. (2000). Racial and ethnic disparities in faculty promotion in academic medicine. JAMA.

[4] Peterson N.B., Friedman R.H., Ash A.S., Franco S., Carr P.L. (2004). Faculty self-reported experience with racial and ethnic discrimination in academic medicine. J. Gen. Intern. Med..

[5] Price E.G., Gozu A., Kern D.E., Powe N.R., Wand G.S., Golden S., Cooper L.A. (2005). The role of cultural diversity climate in recruitment, promotion, and retention of faculty in academic medicine. J. Gen. Intern. Med..

[6] Hayes B. (2008). Increasing the Representation of Underrepresented Minority Groups. Am. J. Pharm. Educ..

[7] Chisholm-Burns M.A., Spivey C.A., Billheimer D., Schlesselman L.S., Flowers S.K., Hammer D., Engle J.P., Nappi J.M., Pasko M.T., Ross L.A. (2012). Multi-Institutional Study of Women and Underrepresented Minority Faculty. Am. J. Pharm. Educ..

[8] Guide T.C.U. (2011). University Subject Tables 2011: Pharmacology & Pharmacy. http://www.thecompleteuniversityguide.co.uk/league-tables/rankings?s=Pharmacology+%26+Pharmacy&y=2011.

[9] Ouyang D. (2012). How to help first-year pharmacy students to gain the big picture. J. Asian Assoc. Sch. Pharm..

[10] Kaplan S.E., Raj A., Carr P.L., Terrin N., Breeze J.L., Freund K.M. (2018). Race/Ethnicity and Success in Academic Medicine: Findings From a Longitudinal Multi-Institutional Study. Acad. Med..

[11] Vongvanith V.V., Huntington S.A., Nkansah N.T. (2012). Diversity Characteristics of the 2008–2009 Pharmacy College Application Service Applicant Pool. Am. J. Pharm. Educ..

[12] Zeind C.S. (2005). Developing a Sustainable Faculty Mentoring Program. Am. J. Pharm. Educ..

[13] Wutoh A.K., Colebrook M.N., Holladay J.W., Scott K.R., Hogue V.W., Ayuk-Egbe P.B., Lombarbo F.A. (2000). Faculty Mentoring Programs at Schools/Colleges of Pharmacy in the U.S. J. Pharm. Teach..

[14] Hagemeier N.E., Murawski M.M., Popovich N.G. (2013). The Influence of Faculty Mentors on Junior Pharmacy Faculty Members’ Career Decisions. Am. J. Pharm. Educ..

[15] MacKinnon G.E. (2003). An Investigation of Pharmacy Faculty Attitudes Toward Faculty Development. Am. J. Pharm. Educ..

[16] Senlin S., Sanmin H., Xuefeng X. (2010). To strengthen the Clinical pharmacy education and cultivatethe pharmaceutical care talents. Pharm. Educ..

[17] Liang H., Zhang X. (2011). Inspiration of Clinical Pharmacy Education Model Overseas to Chinese Pharmacy Education. Med. Soc..

[18] Åkerlind G.S. (2005). Academic growth and development-How do university academics experience it?. High. Educ..

